# Lower Initial Insulin-like Growth Factor-Binding Protein-3 Concentrations May Reflect Immune Suppression and Predict Increased Risk of Sepsis-Related Mortality

**DOI:** 10.3390/ijms26146549

**Published:** 2025-07-08

**Authors:** Filippo Mearelli, Alessio Nunnari, Federica Chitti, Annalisa Rombini, Alessandra Macor, Donatella Denora, Luca Messana, Marianna Scardino, Ilaria Martini, Giulia Bolzan, Francesca Spagnol, Chiara Casarsa, Nicola Fiotti, Verena Zerbato, Stefano Di Bella, Carlo Tascini, Filippo Giorgio Di Girolamo, Mariella Sturma, Venera Costantino, Gianni Biolo

**Affiliations:** 1 Unit of Internal Medicine, Clinica Medica, Department of Medical Surgical and Health Sciences, University of Trieste, Strada di Fiume 447, 34100 Trieste, Italy; 2 Infectious Diseases Unit, Clinical Departement of Medical, Surgical, and Health Sciences, University Hospital (ASUGI), Piazzale dell’Ospedale n° 1, 34100 Trieste, Italy; 3 Infectious Diseases Unit, University Hospital (ASUFC), Via Pozzuolo n° 330, 33100 Udine, Italy; 4 Microbiology Unit, University Hospital (ASUGI), Strada di Fiume n° 447, 34137 Trieste, Italy

**Keywords:** procalcitonin, interleukin-6, lactate, mid-regional pro-adrenomedullin, sepsis, immune suppression, gut dysfunction, insulin-like growth factor-binding protein-3, insulin-like growth factor

## Abstract

Insulin-like growth factor-binding protein-3 (IGFBP-3) plays a vital role in cellular growth, development, and survival. Incorporating IGFBP-3 into baseline prognostic evaluations may enhance the prediction of mortality in patients with sepsis. In this study, serum levels of IGFBP-3, C-reactive protein, procalcitonin, lactate, interleukin-6, and mid-regional pro-adrenomedullin were measured upon admission to the internal medicine unit (IMU) in 139 patients with microbiologically confirmed sepsis. The objectives were as follows: (1) to classify septic patient phenotypes based on optimal thresholds of independent prognostic biomarkers and (2) to evaluate whether these biomarkers improve the predictive accuracy of a clinical model (Model 1), which includes the clinical predictors of 1-year mortality. Age, sequential organ failure assessment (SOFA) score, multiple sources of infection, and IGFBP-3 levels independently predicted 1-year mortality. Patients with IGFBP-3 levels below 10.64 had significantly lower median body temperature (*p* = 0.008), reduced lymphocyte count (*p* = 0.001), and higher 1-year mortality (*p* < 0.001). Model 1 included age, SOFA score, and the presence of multiple sources of sepsis as predictor variables. Model 2 incorporated the same variables as Model 1, with the addition of IGFBP-3 levels. When comparing their prognostic performance, Model 2 demonstrated superior predictive accuracy for mortality at 60, 90, and 365 days following admission to the IMU. Low IGFBP-3 levels at IMU admission are strongly associated with worse outcomes in septic patients, supporting its potential use as a prognostic biomarker.

## 1. Introduction

Sepsis is a life-threatening condition characterized by organ dysfunction resulting from an abnormal host response to infection, and it is a leading cause of morbidity and mortality worldwide [1]. Prompt recognition and stratification of septic patients based on their risk for adverse outcomes are crucial for effective clinical management and resource allocation [2].

Biomarkers such as C-reactive protein (CRP), lactate, procalcitonin (PCT), interleukin-6 (IL-6), and mid-regional pro-adrenomedullin (MR-proADM) are routinely measured at baseline to aid in predicting clinical deterioration and adverse short-term outcomes in septic patients [3,4,5,6]. Sepsis survivors also face a significantly higher risk of long-term mortality compared to the general population, with the greatest risk occurring during the first year after the septic episode, particularly in individuals with pre-existing health conditions or severe disease [7,8]. As a result, sepsis often causes organ damage that can progress to chronic conditions, thereby further increasing the risk of mortality [7,8]. Additionally, sepsis weakens the immune system, making survivors more vulnerable to infections, which may lead to rehospitalizations and higher rates of subsequent mortality [9]. While CRP, lactate, PCT, IL-6, and MR-proADM offer distinct advantages for predicting unfavorable short-term outcomes in sepsis, their comparative and/or combined effectiveness in predicting long-term mortality has yet to be fully explored [3,4,5,6].

Insulin-like growth factor-binding protein-3 (IGFBP-3) has emerged as a potential prognostic marker in septic patients in the intensive care unit (ICU) [10,11]. However, its ability to predict long-term mortality remains unclear in both ICU and non-ICU settings. Understanding how the prognostic variables and biomarkers currently used in clinical practice, along with IGFBP-3, compare with each other, and whether combining them could improve the prediction of 1-year mortality, is crucial. This knowledge is essential for advancing precision medicine in both sepsis and post-sepsis care [3,4,5,6,9].

Therefore, at baseline, in patients with microbiologically documented sepsis, we aimed to achieve the following:

- Assess the prognostic performance of CRP, lactate, PCT, IL-6, MR-proADM, and IGFBP-3 levels in predicting 1-year mortality.
- Identify the independent predictors of 1-year mortality.
- Describe the characteristics of septic patient phenotypes, classified based on the levels of biomarkers shown to be independent predictors of 1-year mortality.
- Derive two multivariable models based on the independent predictors of 1-year mortality: one without biomarkers (Model 1) and one with biomarkers (Model 2).
- Compare the prognostic performance of Model 1 and Model 2 over time.

This prospective study was carried out in the internal medicine department of the University Hospital of Trieste, Italy.

## 2. Results

A total of 334 patients with suspected sepsis were eligible for inclusion in this study. Patients diagnosed with sepsis mimickers (*n* = 55 [16%]) and those with clinically documented sepsis (*n* = 140 [42%]) were excluded from the analysis. The baseline characteristics of the remaining 139 patients with microbiologically documented sepsis (42% of the total cohort) are detailed in Table 1.

### 2.1. Prognostic Performance of the SOFA Score and Serum Biomarkers in Predicting 1-Year Mortality

The SOFA score showed an area under the receiver operating characteristic curve (AUROC) of 0.66 (95% CI: 0.57–0.75) for predicting 1-year mortality. The AUROCs for the serum biomarkers were as follows: CRP, 0.56 (95% CI: 0.46–0.65); PCT, 0.51 (95% CI: 0.41–0.60); MR-proADM, 0.64 (95% CI: 0.55–0.73); IL-6, 0.56 (95% CI: 0.47–0.66); lactate, 0.57 (95% CI: 0.48–0.67); and IGFBP-3, 0.70 (95% CI: 0.61–0.78).

### 2.2. Independent Predictors of 1-Year Mortality

Patients who did not survive within 1 year of IMU admission had significantly higher median CCI scores (4 (3–6)) and SOFA scores (4 (3–6)), as well as significantly higher levels of MR-proADM (3.2 (2.15–4.8) nmol/L) and lower concentrations of IGFBP-3 (37,652 (25,000–51,142) pg/mL) at baseline, compared to those who survived (CCI: 3 (2–5), *p* = 0.007; SOFA score: 3 (2–4), *p* < 0.001; MR-proADM: 2 (1.23–3.85) nmol/L, *p* = 0.005; IGFBP-3: 55,155 (34,969–84,575) pg/mL, *p* < 0.001, respectively). Multiple sources of sepsis were more common in non-survivors (14 [20%]) than survivors (2 [3%]; *p* = 0.003). The percentage of bloodstream infections was higher in survivors (44 [47%]) compared to non-survivors. In addition to the CCI, SOFA score, MR-proADM, IGFBP-3, multiple sources of sepsis, and bloodstream infections, age (*p* = 0.074) and polymicrobial etiology of sepsis (*p* = 0.097) were also included in the Cox regression analysis. The independent predictors of 1-year mortality from IMU admission (Table 2) included age (HR 1.03 [95% CI: 1.00–1.106]; *p* = 0.013; AUROC = 0.59 [0.49–0.68]), SOFA score (HR 1.10 [95% CI: 1.01–1.21]; *p* = 0.039; AUROC = 0.66 [0.57–0.75]), multiple sources of sepsis (HR 3.24 [95% CI: 1.74–5.98]; *p* < 0.001; AUROC = 0.58 [0.49–0.68]), and IGFBP-3 (HR 0.53 [95% CI: 0.34–0.83]; *p* = 0.005; AUROC = 0.70 [0.61–0.78]).

**Figure 1:**
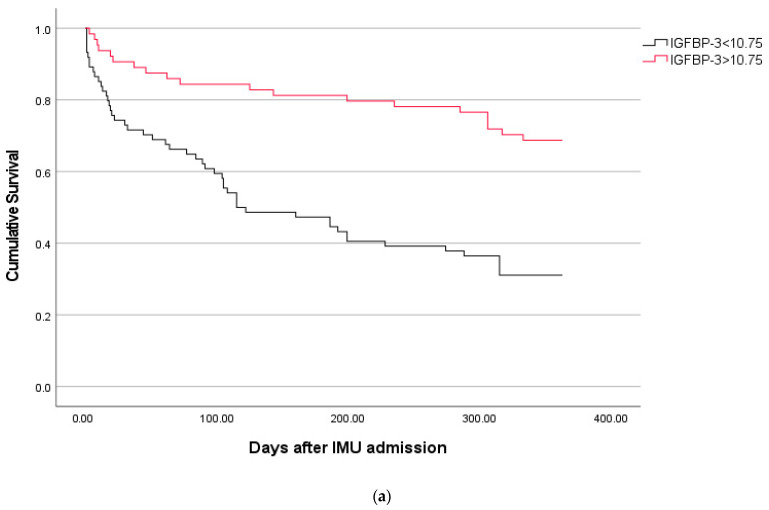

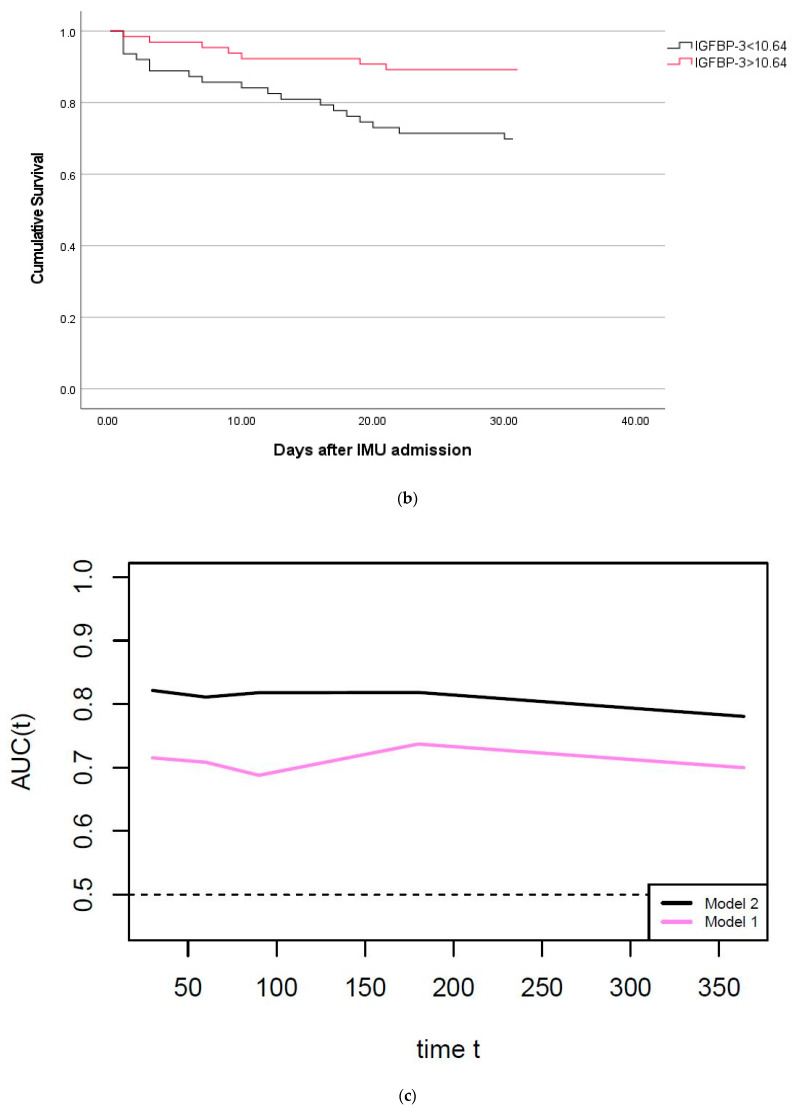
(**a**) Kaplan–Meier curves of freedom from 1-year mortality for patients grouped according to the best prognostic cut-off of insulin-like growth factor-binding protein-3 levels. **Abbreviations:** IGFBP-3 = insulin-like growth factor-binding protein-3 (expressed as Log levels), IMU = internal medicine unit. **Legend:** Log-rank < 0.001. (**b**) Kaplan–Meier curves of freedom from 30-day mortality for patients grouped according to the best prognostic cut-off of insulin-like growth factor-binding protein-3 levels. **Abbreviations:** IGFBP-3 insulin-like growth factor-binding protein-3 (expressed as Log levels), IMU = internal medicine unit. **Legend:** Log-rank = 0.007. (**c**) Time-dependent receiver operating characteristic curves of Model 1 and Model 2 for predicting mortality up to 1 year from internal medicine unit admission. **Abbreviations:** AUC = area under the receiver operating characteristic curve. **Legend:** Model 1 includes age, SOFA score, and multiple sources of sepsis. Model 2 includes age, SOFA score, multiple sources of sepsis, and insulin-like growth factor-binding protein-3 levels expressed as Log levels. Time is expressed in days from internal medicine unit admission.

The optimal cut-off for IGFBP-3 in predicting 1-year mortality was 10.75. The Kaplan–Meier analysis revealed a significant association between this IGFBP-3 cut-off and 1-year mortality (Log-rank *p* < 0.001; Figure 1a).

### 2.3. Phenotypes of Septic Patients According to the Best Prognostic Cut-Off of IGFBP-3 for Predicting 30-Day Mortality

The best cut-off for IGFBP-3 levels in predicting 30-day mortality was identified as 10.64. The Kaplan–Meier analysis demonstrated a significant association between this IGFBP-3 cut-off and 30-day mortality (Log-rank *p* = 0.007; Figure 1b).

Patients with IGFBP-3 levels above (*n* = 72) and below (*n* = 67) 10.64 did not differ significantly in terms of sex (*p* = 0.231), median age (*p* = 0.808), or median CCI (*p* = 0.091). However, chronic liver disease was more common in subjects with lower IGFBP-3 levels (*p* = 0.018; Table 3).

The median SOFA score was 3 (2–5) for individuals with IGFBP-3 levels > 10.64 and 4 (3–6) for those with levels < 10.64 (*p* = 0.040; Table 3). Median values for white blood cell count, CRP, AST, ALT, total bilirubin, creatinine, INR, PCT, MR-proADM, and lactate did not significantly differ between the two phenotypes. Patients with IGFBP-3 levels < 10.64 had a lower median body temperature (37 (36–38) °C) and lymphocyte count (0.86 [0.35–1.28] × 10^9^/L) at baseline compared to those with IGFBP-3 levels > 10.64 (38 [37–38.1] °C; *p* = 0.008 and 1.02 [0.68–1.7] × 10^9^/L; *p* = 0.007, respectively; Table 3). Severe immune suppression (defined as a lymphocyte count <0.5 × 10^9^/L) was more common in the low IGFBP-3 phenotype (*n* = 24 [36%]) than in the high IGFBP-3 phenotype (*n* = 5 [7%]; *p* < 0.001). Median concentrations of IL-6 were significantly higher in subjects with IGFBP-3 levels < 10.64 (305 (76–1004) pg/mL) than in those with IGFBP-3 > 10.64 (107 (37–341) pg/mL; *p* = 0.004; Table 3). There were no significant differences between the two phenotypes in terms of infection source (multiple vs. single source; *p* = 0.290), respiratory vs. non-respiratory source (*p* = 0.178), bacterial vs. non-bacterial etiology (*p* = 0.259), Gram-positive vs. Gram-negative etiology (*p* = 0.421), or bloodstream vs. non-bloodstream infections (*p* = 0.232). However, polymicrobial sepsis was more common in patients with IGFBP-3 levels < 10.64 (*n* = 20 [30%]) compared to those with higher levels (*n* = 9 [13%]; *p* = 0.013; Table 3). Notably, all patients with bacteremia due to Enterococci from the gastrointestinal tract (*n* = 2 [100%]) were part of the low IGFBP-3 phenotype.

### 2.4. Comparison of Prognostic Performance Between Model 1 and Model 2

Model 1 included age, SOFA score, and multiple sources of sepsis. Model 2 incorporated these same variables with the addition of IGFBP-3. The AUROCs for predicting 1-year mortality were 0.70 (95% CI: 0.61–0.79) for Model 1 and 0.78 (95% CI: 0.70–0.86) for Model 2. Time-dependent ROC curves for both models are illustrated in Figure 1c.

Model 2 demonstrated superior prognostic performance compared to Model 1 at all assessed time points following IMU admission, with AUROC values of 0.82 vs. 0.72 at 30 days (*p* = 0.052), 0.81 vs. 0.71 at 60 days (*p* = 0.043), 0.82 vs. 0.69 at 90 days (*p* = 0.006), 0.82 vs. 0.74 at 180 days (*p* = 0.060), and 0.78 vs. 0.70 at 365 days (*p* = 0.042), as shown in Table 4.

## 3. Discussion

The main findings of this study, conducted in internal medicine patients with microbiologically documented sepsis, are as follows: (1) commonly used baseline biomarkers—CRP, lactate, PCT, IL-6, and MR-proADM—demonstrate limited prognostic accuracy for predicting 1-year mortality; (2) IGFBP-3 is the only biomarker independently associated with 1-year mortality; (3) lower baseline levels of IGFBP-3 identify a subgroup of septic patients with greater immunosuppression and a higher risk of both short- and long-term adverse outcomes; and (4) the inclusion of IGFBP-3 significantly improves the prognostic accuracy of a clinical model based on independent predictors of 1-year mortality at multiple time points following admission to IMU.

The prognostic value of biomarkers commonly used in clinical practice has predominantly been evaluated in relation to short-term outcomes, such as 30-day mortality [3,4,5,6]. C-reactive protein (CRP), a general marker of inflammation, remains widely utilized for sepsis prognostication and monitoring treatment response. Lactate, an established indicator of tissue hypoperfusion, is frequently used to assess severity and predict mortality in septic patients. Procalcitonin (PCT), a biomarker more specific to bacterial infections, has shown limited utility in predicting outcomes in sepsis. Elevated levels of interleukin-6 (IL-6) are associated with disease severity, organ dysfunction, and increased risk of death. Mid-regional pro-adrenomedullin (MR-proADM), a stable precursor fragment of adrenomedullin, reflects endothelial dysfunction and vascular integrity—both of which are severely compromised in sepsis [3,4,5,6].

Insulin-like growth factor-binding proteins (IGFBPs) are a group of secreted proteins that regulate the bioavailability and activity of insulin-like growth factors (IGFs) by binding them with high affinity [12,13,14]. Six IGFBPs have been identified and chemically characterized. Among them, IGFBP-3 is the most abundant in circulation, binding up to 95% of IGF-1 and IGF-2 in a ternary complex that includes an acid-labile subunit, thus serving as a major reservoir for circulating IGFs. Unlike other IGFBPs, IGFBP-3 levels remain relatively stable over 24 h. IGFBP-3 exerts IGF-dependent effects modulating cell proliferation, differentiation, and inhibition of apoptosis via the IGF-1 receptor. However, accumulating evidence also supports significant IGF-independent roles of IGFBP-3, particularly in regulating cell growth, survival, and apoptosis [12,13,14]. Dysregulation of these processes can exacerbate organ dysfunction and contribute to mortality in sepsis. Animal models of endotoxemia and clinical studies have consistently shown that sepsis reduces circulating levels of both IGF-1 and IGFBP-3, as well as their hepatic gene expression [15,16,17,18,19,20]. Inducible nitric oxide synthase (iNOS)-mediated overproduction of nitric oxide (NO) has been implicated in suppressing the IGF-1/IGFBP-3 axis [17]. Moreover, NO is known to contribute to cellular toxicity in sepsis, and surrogate markers of NO bioavailability have been correlated with disease severity and prognosis [17,21].

To date, only two clinical studies have evaluated IGFBP-3 as a prognostic biomarker in adult patients with sepsis [10,11]. Papastathi et al. reported an inverse association between IGFBP-3 levels and short-term adverse outcomes, suggesting its potential prognostic value [11]. Conversely, Schuetz et al. found no significant relationship between lower IGFBP-3 concentrations and mortality risk [10]. In the present study, we demonstrated that IGFBP-3 levels below 10.64 were significantly associated with increased 30-day mortality among patients with microbiologically documented sepsis (Log-rank *p* = 0.007). These conflicting findings across studies may be explained by variations in study populations, including differences in age, comorbidity burden, illness severity, and infection characteristics.

While many individuals recover from sepsis, studies have shown that survivors remain at increased risk of death in the months following the acute episode [7,8]. Recent ICU-based research reports a wide range of 1-year mortality rates among sepsis patients, from 12% to 66% [7,8]. The elderly are the most frequently affected demographic [22]. Internal medicine patients, who are typically older and burdened with multiple comorbidities, reduced functional capacity, and immune senescence, are especially vulnerable, facing an elevated risk of both short- and long-term adverse outcomes [22]. However, data on the long-term prognosis of septic patients managed outside the ICU, particularly in internal medicine settings, remain scarce. In our cohort, the 1-year mortality rate was 51%, aligning with rates observed in ICU populations [7,8,22]. The prognostic performance of commonly used biomarkers—CRP, lactate, PCT, IL-6, and MR-proADM—for predicting 1-year mortality was limited in this study, with all median AUROC values below 0.65. To our knowledge, no prior studies have assessed the prognostic utility of IGFBP-3 for long-term outcomes in sepsis. Among the six biomarkers evaluated, only IGFBP-3 emerged as an independent predictor of 1-year mortality in the Cox regression analysis. Notably, a single baseline IGFBP-3 measurement demonstrated the same AUROC (0.70) for 1-year mortality as the combined predictive performance of Model 1, which included other independent clinical predictors such as age, SOFA score, and multiple sources of sepsis.

Our findings provide potential explanations for the inverse relationship between serum IGFBP-3 levels and both short- and long-term adverse outcomes in internal medicine patients with sepsis. One plausible mechanism underlying this association is heightened baseline immune suppression, which may increase susceptibility to unresolved primary infections, secondary infections, or impaired recovery, ultimately contributing to elevated short- and long-term mortality [9,22]. In our cohort, patients with IGFBP-3 levels below 10.64 exhibited significantly lower median lymphocyte counts and a higher prevalence of severe lymphocytopenia at baseline compared to those with higher IGFBP-3 levels. This observation aligns with preclinical studies demonstrating that (1) nearly all immune cells, including T and B lymphocytes, are responsive to the stimulatory effects of IGF-1, and (2) IGFBP-3 directly promotes lymphocyte production [23,24,25,26]. Furthermore, patients with IGFBP-3 < 10.64 also had lower median body temperatures at admission, suggesting a blunted systemic inflammatory response and supporting the hypothesis of more pronounced immune suppression in this phenotype. Notably, lower body temperature has been associated with poorer outcome in septic patients [27]. IGFBP-3 has been shown to exert important functions beyond its classical role in modulating IGF-1 activity in in vivo models [28]. In sepsis, IGFBP-3 demonstrates IGF-1-independent effects, including the modulation of immune responses, regulation of apoptosis, and influence on inflammation [13]. These IGF-1-independent actions of IGFBP-3 may help explain the immune suppression and increased severity observed in septic patients with low IGFBP-3 levels.

Beyond its immunomodulatory role, the IGF-1/IGFBP-3 axis has also been implicated in gut barrier function. Apoptosis of intestinal epithelial cells is a key contributor to gut dysfunction in sepsis, leading to increased intestinal permeability, impaired nutrient absorption, and enhanced bacterial translocation [28]. This translocation facilitates systemic dissemination of enteric pathogens and endotoxins, promoting multiorgan failure and increased mortality risk. The IGF-1/IGFBP-3 complex has been shown to exert anti-apoptotic effects on small intestinal epithelial cells [29,30,31,32,33], and lower IGFBP-3 levels have been associated with disease activity in inflammatory bowel disease [29,30]. Animal studies further support a link between decreased IGF-1/IGFBP-3 expression and increased bacterial translocation [31,32,33]. In our study, all cases of Enterococcus bacteremia—a recognized marker of gastrointestinal barrier dysfunction in sepsis [34]—occurred in patients with IGFBP-3 levels below 10.64, reinforcing the potential association between low IGFBP-3 and gut-derived septic complications.

Although this was an observational study, our findings underscore the potential clinical utility of measuring IGFBP-3 levels in sepsis patients admitted to internal medicine units. Notably, patients with IGFBP-3 levels below 10.64 experienced approximately double the 30-day and 1-year mortality rates compared to those with higher levels. This suggests that IGFBP-3 could serve as a valuable biomarker for the early identification of individuals at higher risk of clinical deterioration. Such early risk stratification could facilitate timely decisions regarding closer monitoring, more aggressive management strategies, or preemptive transfer to the ICU. Post-sepsis care—including long-term monitoring, rehabilitation, and the management of comorbid conditions—has been shown to reduce the risk of death and hospital readmission among survivors 35. Our data suggest that baseline IGFBP-3 levels may help identify patients who would benefit from such targeted interventions. The prognostic value of IGFBP-3 became more evident when integrated into a multivariable prognostic model. We developed two models: Model 1, which included all clinical variables independently associated with 1-year mortality, and Model 2, which added IGFBP-3 to Model 1. Model 2 demonstrated the highest predictive accuracy for 1-year mortality (AUROC = 0.78). While the addition of IGFBP-3 led to only a borderline improvement in early prognostication (e.g., 30-day AUROC, *p* = 0.052), it significantly enhanced the performance of Model 1 at later time points. Specifically, Model 2 outperformed Model 1 at 60, 90, and 365 days post-admission, consistently achieving AUROC values above 0.77—indicating excellent predictive capability.

Research is increasingly focused on identifying biomarkers that reflect key pathophysiological mechanisms and can guide patient selection for predictive enrichment in clinical trials of targeted therapies [3,4,5,6]. IGFBP-3 shows promise in guiding the selection of septic patients for interventional trials aimed at reversing immune suppression. Personalized medicine in sepsis management involves measuring prognostic biomarkers that provide insight into specific pathway activities, coupled with targeted interventions to modulate these pathways [3,4,5,6]. IGFBP-3 may also represent a potential therapeutic target in future sepsis research [13]. Several preclinical studies have demonstrated the feasibility and benefits of modulating IGF-1/IGFBP-3 expression in acute pathologies, including sepsis. For example, in acute endotoxemia models, aminoguanidine (an iNOS inhibitor) [17] and dexamethasone [35] prevented the suppressive effects of lipopolysaccharide on IGF-1 and IGFBP-3 levels and expression. IGF-1 replacement was shown to protect Kupffer cells from apoptosis and improve survival in mice by enhancing bacterial clearance [36]. Additionally, recombinant IGFBP-3 attenuated acute lung injury [37]. In rats with thermal injury and endotoxemia, treatment with IGF-1/IGFBP-3 improved villous height and cell count per villus by reducing epithelial apoptosis and promoting proliferation [32,33]. Furthermore, our findings show that lower IGFBP-3 levels are associated with elevated IL-6 concentrations and more severe lymphocytopenia, suggesting a possible synergistic role of IGFBP-3 and IL-6 in exacerbating immune suppression. In COVID-19 patients, IL-6 has been shown to drive T cell death, contributing to lymphopenia [38]. Future studies should explore the mechanistic interaction between IGFBP-3 and IL-6 and evaluate combined therapeutic strategies targeting both pathways.

This study has several limitations. Liver synthesis is the primary source of circulating IGFBP-3 [39]. Notably, chronic liver disease was more prevalent among patients with lower IGFBP-3 levels in our cohort. Although standard liver function tests (AST, ALT, INR, and bilirubin) were not significantly different between groups, we cannot exclude the possibility that lower IGFBP-3 concentrations may, in part, reflect subclinical hepatic dysfunction at the time of IMU admission [39]. IGFBP-3 levels may reflect proteolytic fragments, as protease activity against IGFBP-3 has been observed in critically ill patients with various diagnoses [19,40]. While we did not measure protease levels in this study, the clinical relevance of these findings remains uncertain [19,40]. Another important limitation of this research is that IGFBP-3 is not currently measurable at the bedside in clinical practice. Thus, we cannot conclusively support IGFBP-3 as a surrogate marker for gastrointestinal dysfunction in sepsis since we did not assess clinical variables of gut function at baseline [34], the number of bacteremia caused by Enterococci from the gastrointestinal tract (cases [34]) was small, and there were no cases of bacteremia caused by coagulase-negative staphylococci (controls [34]). Although we did not directly measure IGF-1 levels, previous studies have shown that IGFBP-3 is the strongest determinant of IGF-1 concentrations, accounting for most of the variance in IGF-1 levels [12,13,14]. Thus, we analyzed six prognostic biomarkers at the time of IMU admission. While single measurements can provide valuable information, they may overlook important dynamic changes in a patient’s condition [41,42,43]. Repeated testing may offer a more effective approach for managing sepsis by capturing these fluctuations. Lastly, the prognostic performance of the predictive models we developed should be validated in external cohorts to confirm their accuracy and generalizability [41].

## 4. Materials and Methods

This study was conducted between 31 December 2015 and 31 December 2016. Patients with a SOFA score ≥ 2 and who had blood cultures taken and received antibacterial therapy during their stay in the emergency department (suspected sepsis) were eligible for inclusion. Upon admission to the internal medicine unit (IMU), general patient information was collected, including sex, age, and the Charlson Comorbidity Index (CCI). Baseline vital signs recorded included body temperature and the parameters necessary to calculate the SOFA score. Initial laboratory results included leukocyte count, lymphocyte count, glucose, creatinine, sodium, potassium, AST, ALT, bilirubin, and INR. Prognostic biomarkers available at the time of IMU admission included CRP, PCT, lactate, IL-6, and MR-proADM.

At the end of clinical follow-up, patients with suspected sepsis were classified as having either a confirmed infection (sepsis) or an acute illness that mimicked infection (sepsis mimickers) by the data review committee. Patients with sepsis were further categorized as having either a single or multiple sources of infection. Single-source infections were classified as respiratory or non-respiratory. Septic patients were also grouped based on whether the pathogen was identified: microbiologically documented sepsis or clinically documented sepsis. Microbiologically documented infections were categorized as monomicrobial if caused by a single pathogen or polymicrobial if multiple pathogens were identified. For monomicrobial cases, the etiology was categorized as bacterial or non-bacterial. In bacterial sepsis, pathogens were further classified by Gram stain (Gram positive or Gram negative). Immune suppression was defined as a lymphocyte count of less than 1000 × 10^9^/L, and severe immune suppression as less than 500 × 10^9^/L. Mortality was monitored from the time of IMU admission for up to one year. Mortality at 30 days and 1 year was used to assess short-term and long-term outcomes, respectively.

### 4.1. Serum Biomarkers

Serum levels of CRP, lactate, PCT, IL-6, and MR-proADM were measured within six hours of admission to the IMU in all patients with suspected sepsis. Blood samples for IGFBP-3 determination were also collected within six hours of IMU admission. Serum aliquots were frozen within one hour of collection and stored at −80 °C. IGFBP-3 levels were measured using a commercial enzyme-linked immunosorbent assay (ELISA) kit (Thermo Fisher, Invitrogen; Dreieich, Germany; EHIGFBP3) in thawed serum samples from patients diagnosed with microbiologically documented sepsis at the end of clinical follow-up. PCT and MR-proADM concentrations were determined in serum with a fully automated Brahms Kryptor Gold Analyzer (Thermo Scientific, Dreieich, Germany). CRP, lactate, and IL-6 levels were measured using a fully automated Elecsys system on the cobas e801 platform (Roche Diagnostics, Basel, Switzerland).

This study was approved by the Ethics Committee of the University of Trieste (Report No. 39) and conducted in accordance with the Declaration of Helsinki. Informed consent was obtained from each participant or their legally authorized representative before data collection.

### 4.2. Statistical Analysis

Clinical variables and biomarkers associated with non-survivor status (*p* ≤ 0.1) at 1 year from IMU admission were considered potential candidates for inclusion in the Cox regression analysis. Hazard ratios (HRs) and 95% confidence intervals (CIs) were calculated for all clinical variables and biomarkers identified as independent predictors of 1-year mortality. The optimal prognostic cut-off values for each biomarker, for both 30-day and 1-year mortality, were determined based on the values that yielded the highest specificity for predicting non-survivor status. Patients with microbiologically documented sepsis were then categorized according to the best 30-day mortality cut-off for each independent biomarker. Clinical characteristics of the resulting phenotypes (patients above or below the cut-off) were subsequently compared. Two prognostic models were developed to predict 1-year mortality. Model 1 included only the independent clinical variables, while Model 2 incorporated both clinical variables and biomarkers identified as independent predictors. The discriminatory power of the clinical variables, biomarkers, and both models in predicting 1-year mortality was assessed using C statistics (area under the receiver operating characteristic curve, AUROC). The DeLong test was used to compare the AUROCs of the models at various time points following IMU admission. Survival analyses were conducted using the Kaplan–Meier method, with group comparisons performed using the Log-rank test. All *p*-values were two-sided, with statistical significance set at <0.05. Statistical analyses were performed using the R statistical computing environment (version 4.2.3) and SPSS (version 28.0.0).

## 5. Conclusions

IGFBP-3 is an independent predictor of 1-year mortality in internal medicine patients with microbiologically confirmed sepsis. Lower baseline levels of IGFBP-3 identify a phenotype of septic patients who experience more severe immune suppression and are at higher risk for both 30-day and 1-year mortality. Finally, IGFBP-3 enhances the prognostic accuracy of a predictive model based on clinical variables that have been established as independent predictors of 1-year mortality at 60, 90, and 365 days following IMU admission.

## Figures and Tables

**Table 1:** Patients with sepsis microbiologically documented analyzed in the study: summary of clinical characteristics.

Characteristics	*n* = 139 (100)
Female	55 (40)
Median age	79 (72–86)
Median Charlson Comorbidity Index	3 (2–5)
Median SOFA score at IMU admission	3 (2–5)
**Biomarkers**	
Median CRP (mg/L)	128 (51–240)
Median PCT (ng/mL)	1.8 (0.33–11.4)
Median MR-proADM (nmol/L)	2.8 (1.71–4.37)
Median IL-6 (pg/mL)	186.6 (43.8–605.2)
Median lactate (mg/dl)	15 (11–23)
Median IGFBP-3 (pg/mL)	42,150 (29,478–66,077)
**Source of infection**	
Multiple	16 (12)
Respiratory	36 (26)
**Etiology of infection**	
Monomicrobial	110 (79)
-Gram +	39 (35)
-Gram –	68 (62)
-Non bacterial	3 (3)
Polymicrobial	29 (21)
Bloodstream infections	77 (55)
**Mortality**	
30 days	26 (19)
1 year	71 (51)

**Abbreviations:** SOFA = sequential organ failure assessment, CRP = C-reactive protein, PCT = procalcitonin, MR-proADM = mid-regional pro-adrenomedullin, IL-6 = interleukin-6, IGFBP-3 = insulin-like growth factor-binding protein-3. **Legend:** Categorical data are presented as absolute frequencies and percentages, while continuous data are shown as medians with interquartile ranges. Mortality rates at 30 days and 1 year following ICU admission were 19% (*n* = 26) and 51% (*n* = 71), respectively.

**Table 2:** Independent predictors of 1-year mortality: regression coefficients, levels of significance, hazard ratios, and area under the receiver operating characteristic curves.

Predictor	*B*	*p*	Hazard Ratio (95% CI)	AUROC (95% CI)
Age	0.037	0.013	1.03 (1–1.06)	0.59 (0.49–0.68)
SOFA score	0.10	0.039	1.10 (1–1.21)	0.66 (0.57–0.75)
Multiple source	1.174	<0.001	3.24 (1.74–5.98)	0.58 (0.49–0.68)
IGFBP-3 ^	−0.622	0.005	0.53 (0.34–0.83)	0.70 (0.61–0.78)

**Abbreviations:** CI = confidence interval, AUROC = area under the receiver operating characteristic curve, SOFA = sequential organ failure assessment, IGFBP-3 = insulin-like growth factor-binding protein-3. **Legend:** Covariates: Charlson Comorbidity Index, mid-regional pro-adrenomedullin, polymicrobial sepsis, and bloodstream infections. ^ Expressed as Log levels.

**Table 3:** Characteristics of patients according to the best prognostic cut-off of insulin-like growth factor-binding protein-3 for predicting 30-day mortality: significant differences between the two phenotypes.

Characteristics	IGFBP-3 < 10.64 * *n* = 67	IGFBP-3 > 10.64 * *n* = 72	*p*
Chronic liver disease	13 (19)	4 (6)	0.018
Median body temperature (°C)	37 (36–38)	38 (37–38.1)	0.008
Median SOFA score at IMU admission	4 (3–6)	3 (2–5)	0.040
**Laboratory available at IMU admission**			
Median lymphocyte count (×10^9^/L)	0.86 (0.35–1.28)	1.02 (0.68–1.7)	0.007
-Lymphocyte count <0.5 (×10^9^/L)	24 (36)	5 (7)	<0.001
Median serum levels of IL-6 (pg/mL)	305 (76–1004)	107 (37–341)	0.004
**Etiology of infection**			
Polymicrobial sepsis	20 (30)	9 (13)	0.013
**Outcome**			
30-day mortality	19 (28)	7 (10)	0.008
1-year mortality	46 (69)	25 (35)	<0.001

**Abbreviations:** IGFBP-3 = insulin-like growth factor-binding protein-3, SOFA = sequential organ failure assessment, IMU = internal medicine unit, IL-6 = interleukin-6. **Legend:** Categorical data are presented as absolute frequencies and percentages, while continuous data are shown as medians with interquartile ranges. * Expressed as Log levels.

**Table 4:** Comparison of the areas under the receiver operating characteristic curves for Model 1 and Model 2 in predicting mortality at various time points from admission to the internal medicine unit.

Models	30 Days AUROC (95% CI)	60 Days AUROC (95% CI)	90 Days AUROC (95% CI)	180 Days AUROC (95% CI)	365 Days AUROC (95% CI)
Model 1: clinical variables ^	0.72 (0.6–0.84)	0.71 (0.57–0.85)	0.69 (0.58–0.79)	0.74 (0.65–0.82)	0.70 (0.61–0.79)
Model 2: clinical variables ^ + IGFBP-3	0.82 (0.74–0.91)	0.81 (0.72–0.90)	0.82 (0.75–0.89)	0.82 (0.75–0.89)	0.78 (0.70–0.86)
*p °*	0.052	0.043	0.006	0.06	0.042

**Abbreviations:** AUROC = areas under the receiver operating characteristic curves, 95% CI = 95% confidence interval, IGFBP-3 = insulin-like growth factor-binding protein-3 levels expressed as Log levels. **Legend:** ^ clinical variables = age, sequential organ failure assessment score, and multiple source of sepsis; ° comparison of discriminating accuracy for death between Model 1 and Model 2 at different times from internal medicine unit admission (De Long test).

## Data Availability

The datasets from this study are available from the corresponding author on request.

## Abbreviations

IMU = internal medicine unit, ICU = intensive care unit, CCI = Charlson Comorbidity Index, SOFA = sequential organ failure assessment, OR = odds ratio, HR = hazard ratio, 95% CI = 95% confidence interval, CRP = C-reactive protein, PCT = procalcitonin, IL-6 = interleukin 6, MR-proADM = mid-regional pro-adrenomedullin, IGFBP-3 = insulin-like growth factor-binding protein, IGFs = insulin-like growth factors, ROC = receiver operating characteristic curve, AUROC = area under the receiver operating characteristic curve, NO = nitric oxide, iNOS = inducible nitric oxide synthase.
